# Computational Analysis of the Potential Impact of MTC Complex Missenses SNPs Associated with Male Infertility

**DOI:** 10.1155/2022/1664825

**Published:** 2022-03-18

**Authors:** Houda Harmak, Hicham Charoute, Salaheddine Redouane, Ouafaa Aniq Filali, Abdelhamid Barakat, Hassan Rouba

**Affiliations:** ^1^Laboratory of Genomics and Human Genetics, 1 Place Louis Pasteur, Institut Pasteur du Maroc, Casablanca, Morocco; ^2^Laboratory of Physiopathology, Molecular Genetics and Biotechnology, Department of Biology, Faculty of Sciences Ain Chock, Hassan II University, Casablanca, Morocco; ^3^Research Unit of Epidemiology, Biostatistics and Bioinformatics, Institut Pasteur du Maroc, Casablanca, Morocco

## Abstract

Meiotic chromosomes endure rapid prophase movements that ease the formation of interhomologue recombination intermediates that drive synapsis, crossing over, and segregation process. To generate these fast moves, the meiotic telomere complex (MTC) enables telomere-inner nuclear membrane attachment during meiotic prophase I and transfers cytoskeletal signals via another complex: the LINC complex. Furthermore, disruption or mutations of any of the MTC genes (TERB1, TERB2, and MAJIN) alters telomere association with the nuclear envelope leading to impairment of homologous pairing and synapsis, a meiotic arrest, and consequently to male infertility. To decipher the effect of TERB1, TERB2, and MAJIN missense mutations on protein structure, stability, and function, different bioinformatic tools were used in this study including VEP, Mutabind2, Haddock, Prodigy, Ligplot, ConSurf, DUET and MusiteDeep. In total, thirty mutations were predicted to be deleterious using VEP web server: seventeen for TERB1, eleven for TERB2, and two for MAJIN. All these single nucleotide polymorphisms were further analyzed and only 11 SNPs (W8R, G25R, P649A, I624T, C618R, F607V, S604G, C592Y, C592R, G187W, and R53C) were found to be the most damaging by at least six software tools and exert deleterious effect on the TERB1, TERB2, and MAJIN protein structures and likely functions. They revealed high conservation, less stability, and having a role in posttranslational modifications. This in silico approach provides information to gain further insights about variants that might affect stability, change binding affinity, and edit protein-protein interactions to facilitate their identification and functional characterization associated with male infertility.

## 1. Introduction

Telomeres have critical meiosis-specific functions in the reduction of chromosome numbers by assuring the integrity of the genome during meiosis. One such function is the attachment of telomeres to trans-nuclear envelope protein complexes that join telomeres to motor proteins in the cytoplasm. The active movement of telomeres and chromosomes during the first meiotic prophase is enabled by these trans-nuclear envelope linkages between telomeres and cytoplasmic motor proteins. Movements of chromosomes/telomeres facilitate the meiotic recombination process and ensure high fidelity pairing of homologous chromosomes which is a prerequisite for their correct segregation during the first meiotic division [1].

Meiosis is a gametogenesis-specific cell division that entails unique chromosomal regulations such as pairing, synapsis, and homologous chromosome recombination. These processes are ensured by the dynamic chromosomal rearrangements that happen during meiotic prophase I which is prolonged and more complicated than mitosis. Indeed, it can last many days and account for up to 90% of the total time spent in meiosis [2]. During meiotic prophase I, chromosomes undergo rapid movements that enhance the constitution of interhomologue recombination intermediates underlying synapsis, crossing over, and segregation processes [3] and ensure the pairing and eventual recombination of homologous chromosomes and the resolving of undesirable entanglements between nonhomologous partners [2].

The recently discovered tripartite complex comprising telomere repeats-binding bouquet formation proteins 1 and 2 (TERB1 and TERB2) and membrane-anchored junction protein (MAJIN) has been demonstrated to be involved in these processes by tethering telomere ends to the nuclear envelope (NE), transmitting cytoskeletal forces via the LINC complex to drive these rapid movements, and assembling of meiotic mammalian telomeres on the NE during prophase I [4].

**Figure 1:**
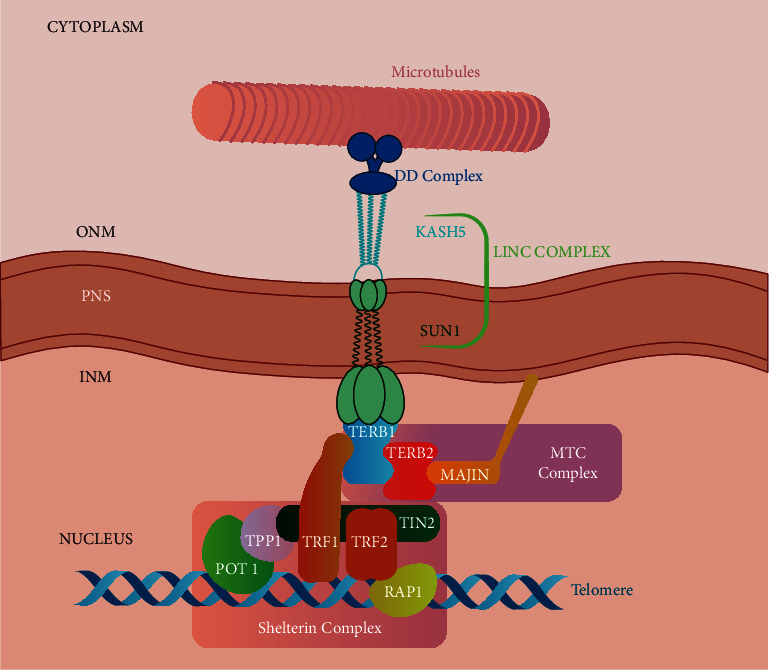
The TERB1-TERB2-MAJIN complex tether telomeres to the nuclear envelope during meiotic prophase I.

In mammals, the meiotic telomere complex (MTC) reuses and integrates the functions of two complexes, Shelterin and LINC, which otherwise play distinct significant roles outside of meiosis [3]. The MTC complex is initially sequestered onto the NE by the membrane-binding activity of MAJIN and then assembles on the telomeres when they approach the nuclear periphery at meiotic entry. The assembly of TERB1, TERB2, and MAJIN onto telomeres is needed for the attachment of the LINC complex proteins (SUN1 and KASH5), and it is required for a stable telomere-NE attachment before the telomere movement (Figure 1) [4].

### 1.1. Identification of Mammalian Shelterin Complex

Mammalian telomeres contain a specific protein complex that accomplishes two functions: protecting the natural chromosome end from all factors of the DNA damage response within the cell and negative regulation of telomerase by segregation of its telomeric DNA substrate. Both functions of this complex refer to the name shelterin [5]. The shelterin complex includes six telomere-specific proteins TRF1, TRF2 (telomeric repeat binding factors family proteins 1 and 2), POT1 (protection of telomeres 1), RAP1 (Repressor/activator protein1), TIN2 (TRF1-interacting protein 2), and TPP1 (TINT1/PTOP/PIP) that protect telomeres from degradation, inhibits unnecessary repair mechanisms, controls telomerase production, and is involved in cellular senescence and age-related pathologies [6, 7].

Because of their TRF homology domains, the shelterin complex's subunits TRF1 and TRF2 perform a fundamental role by recognizing telomeric DNA, and by recruiting the other shelterin constituents onto telomeric DNA [8, 9], where the reformed shelterin core of TRF2-TIN2-TPP1-POT1 has a stoichiometry of 2 : 1 : 1 : 1 in vitro [10]. During prophase, the MTC complex matures, allowing the shelterin/telosome complex to be released from telomeric DNA [7]. Shelterin acts as a telomere recognition protein for the meiotic telomere complex (MTC), (Figure 1) externally connecting it to the LINC complex and allowing them to work together to achieve cytoskeletal attachments of meiotic telomere ends around the nuclear envelope [3].

### 1.2. Identification of Mammalian LINC Complex

Via a perinuclear interaction, SUN1 (Sad1 and UNC84 domain containing 1) and KASH5 (Klarsicht/ANC-1/Syne/homology 5) proteins assemble into the mammalian meiosis specific LINC complex (linker of nucleoskeleton and cytoskeleton), providing the binding sites for telomeres on the inner surface of the nuclear envelope (NE) [11, 12] and procuring the physical linkage to transfer cytoskeletal force transduction from the cytoplasm to the nucleus [13–15]. KASH proteins interact with the cytoskeleton across the outer nuclear membrane, whereas SUN proteins connect with nuclear lamin A and emerin across the inner nuclear membrane [16–19]. In meiotic prophase I, the nuclear lamina endures an important reorganization where LINC complexes link with the meiotic telomere complex (MTC), thanks to movements assured by SUN1 and KASH5 proteins, which interact with microtubules via dynein-dynactin (Figure 1) [16, 20].

### 1.3. The Meiotic Telomere Complex (MTC)

Another protein complex consisting of TERB1 (telomere repeat binding bouquet formation protein 1), TERB2 (telomere repeat binding bouquet formation protein 2), and MAJIN (membrane anchored junction protein) has been demonstrated to build up a second actual linkage for telomere connection to the nuclear envelope by assuring meiotic telomere attachments in mammals [21, 22].

The meiosis-specific telomere regulator TERB1 is a molecular scaffold that simultaneously interacts with SUN1 and meiotic cohesin subunit SA3 through its N-terminal ARM (armadillo) repeat domain and C-terminal Myb domain, respectively, providing telomere attachment to the inner nuclear membrane (INM) and driving the chromosome movement essential for homologous pairing and recombination [21, 22].

Through a region surrounding its TERB2-binding site, TERB1 interacts directly with shelterin constituent TRF1, including a peptide interaction that resembles TIN2 binding to the TRF1 dimeric cleft [4, 23]. Consequently, Long et al. presumed that the disruption of the TERB1-TRF1 interaction impairs telomeric localization of TERB1 and SUN1 in spermatocytes and that the TERB1-TRF1 interface is specific and required for both in vitro and in vivo linking of TERB1 to TRF1 [4].

Therefore, an interaction of the meiotic telomere complex with TRF1 seems to be transient in the cell. MAJIN–TERB2–TERB1 assembles on the nuclear envelope and endures TRF1-dependent recruitment of telomere ends during leptotene and zygotene [4, 22]; CDK activity at that point triggers the relocation of TRF1 to flanking areas in pachytene, with MAJIN–TERB2–TERB1 remaining related with telomere ends [22].

Thus, in mice, knocking out the Terb1 gene disturbs the whole interaction network and affects homolog pairing, synapsis, and recombination, resulting in early spermatogenesis and oogenesis [21]. However, the relevance of each of the TERB1-mediated contacts and their molecular mechanisms in meiosis remain unknowns [4].

MAJIN is a putative transmembrane (TM) protein, located at the inner surface of the NE; it serves as an anchoring component for the inner nuclear membrane through a transmembrane helix at its C-terminus [22]. MAJIN has DNA-binding activity, suggesting that it is involved in the stability of telomere attachment to the inner membrane of the nucleus. MAJIN and TERB1 are physically linked by TERB2, which binds MAJIN through its C-terminus and TERB1 through its N-terminus [4, 22]. Individual disturbance of meiotic telomere complex components causes meiotic arrest in mice, with loss of telomere attachments and chromosomal movements, failure of DNA double-strand break repair, and disrupted synapsis [21, 22]. Accordingly, the disturbance of telomere-NE connection impairs the homologous searching that causes subsequent meiosis defects, including abolished telomere-NE attachment, aberrant homologous pairing, disordered synapsis, and infertility in both sexes [24]. Collectively, these results reveal a linear interaction network within the TERB1, TERB2, and MAJIN proteins that shape together a stable complex that performs a major role in regulating the recruitment of telomeres to the NE (Figure 1) [21–24].

Lately, computational analysis is designed to more accurately predict the impacts of mutations on proteins interactions. By using modern bioinformatics tools as a systematic in silico approach, our study is aimed at identifying the deleterious SNPs in the TERB1, TERB2, and MAJIN genes and predicting their significant pathogenic impact on the functions, stability, and structures of TERB1-TERB2 complex and TERB2-MAJIN complex.

## 2. Materials and Methods

**Figure 2:**
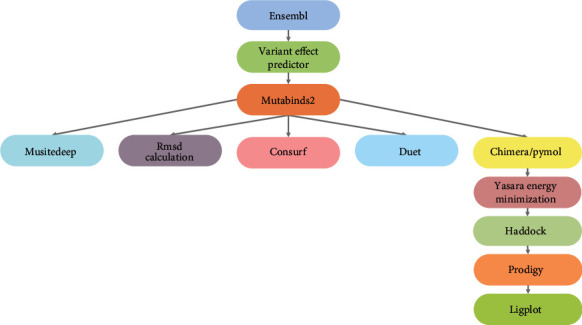
Schematic representation of in silico workflow used in this study.

In this study, we carried out a systematic in silico approach using modern bioinformatics tools to predict deleterious SNPs in the TERB1, TERB2, and MAJIN genes and identify their significant pathogenic impact on the functions and structures of the TERB1, TERB2, and MAJIN proteins (Figure 2).

### 2.1. Retrieval of Variant Dataset

The variant data related to the meiotic telomere complex components (TERB1-TERB2-MAJIN) was retrieved from Ensembl (https://www.ensembl.org): TERB1 (ENST00000433154.6), TERB2 (ENST00000340827.4), and MAJIN (ENST00000301896.6).

### 2.2. Prediction of Mutations Effects

To predict the impact of the retrieved SNPs, the Ensembl Variant Effect Predictor (VEP) was utilized. This tool provides an indication of the effect of the amino acid change using protein biophysical properties [25]. These data can help with the interpretation of protein variants with no associated phenotype or disease data by estimating how deleterious a given mutation may be on the functional status of resulting protein [25]. Scores and predictions are calculated for all possible amino acid substitutions using different algorithms as follows.

The Sorting Intolerant from Tolerant (SIFT) algorithm is predicting whether an amino acid substitution affects a protein function. It takes into account the position of the mutation as well as the type of amino acid change. SIFT assesses the probability that an amino acid at a particular location will be tolerated based on the most frequently tolerated amino acid. The substitution is predicted to be deleterious, if the normalized value is smaller than a cutoff. Scores range from 0 to 1. The smaller the score, the more likely the SNP has damaging effect [26–28].

Polymorphism phenotyping (PolyPhen2) is a tool that predicts the possible impact of amino acid substitutions on the human protein structure and function using structural and comparative evolutionary considerations. PolyPhen2 results are available for human proteins. It classifies the substitution as probably damaging (score = 1 : 0) and possibly damaging or benign (score = 0 : 0) [29].

Other pathogenicity predictor scores such as MutationTaster, MetaSVM, and MetaLR are available for human data via VEP plugins:

MutationTaster is used for a quick assessment of the disease-causing potential of DNA sequence changes. Evolutionary conservation, splice-site changes, protein feature loss, and changes that could affect the amount of mRNA are all examined using MutationTaster, which predicts an alteration as one of the four possible types: “A” (disease causing automatic), “D” (disease-causing), “N” (polymorphism), or “P” (polymorphism automatic) [30, 31];

MetaSVM (SVM (Support Vector Machine)) is a linear model that can be used to solve classification and regression problems. The theory behind SVM is simple: it finds and creates a line (hyperplane) that separates the data into different classes. Prediction using SVM approach in Ensembl database reveals “T” as tolerated or “D” as damaging or deleterious. The score cutoff between “D” and “T” is 0, and higher scores are more deleterious [32];

In MetaLR, to predict the deleteriousness of missense variants, logistic regression (LR) is used to combine nine independent variant deleteriousness scores with allele frequency information. Variants are categorized as “tolerated” or “damaging”; a score between 0 and 1 is also provided where variants with higher scores are more likely to be deleterious [32].

### 2.3. Protein Sequence

**Figure 3:**
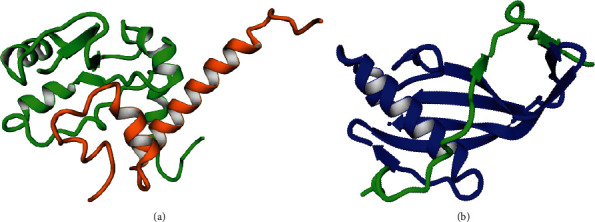
Three-dimensional structures of complexes. (a) TERB1-TERB2 complex. (b) TERB2-MAJIN complex.

The three-dimensional structure of each complex was downloaded from RCSB Protein Data-Bank (https://www.rcsb.org/): The PDB ID for TERB1-TERB2 complex is 6J07 and for TERB2-MAJIN complex is 6GNY (Figure 3).

### 2.4. Prediction of SNP Effects on Protein-Protein Interactions

In order to estimate the impacts of single mutations on protein-protein interactions (PPI), we performed computational analysis on the human crystal structure of TERB1-TERB2 and TERB2-MAJIN complexes using MutaBind2 [33]. This tool compares binding affinity after mutations to predict whether they stabilize or destabilize the PPI by determining the overall change in binding free energies (ΔΔ*G*) by providing the following results: The ΔΔ*G*_bind_ (kcal mol^−1^) which is the predicted change in binding affinity induced by a mutation; in deleterious (yes or no), MutaBind2 server classifies a mutation as deleterious if ΔΔ*G* ≥ 1.5 or ≤−1.5 kcal mol^−1^, ΔΔ*E*_vdw_ is the change of van der Waals interaction energy upon a mutation, ΔΔ*G*_solv_ is the change of polar solvation energy upon mutation, and ΔΔ*G*_fold_ is the change of stability of protein complex upon mutation [33].

### 2.5. Mutagenesis and Energy Minimization

The chains of each complex (A and B of the 6J07 complex and C and D of the 6GNY complex) were separated using the UCSF CHIMERA program [34]. Mutant structures of TERB1, TERB2, and MAJIN proteins were created manually by PyMol software (ver.2.4, Schreödinger) [35]. Then, the energy minimization of structures was performed by YASARA Energy Minimization web server [36].

### 2.6. Molecular Docking Study

The High Ambiguity Driven protein-protein Docking (HADDOCK) web server was employed to perform protein docking. The HADDOCK2.4 uses chemical shift perturbation data resulting from NMR titration experiments, mutagenesis data, and bioinformatics predictions [37]. In the HADDOCK2.4 submission form, we docked the structures of the energy minimized mutated proteins with each other's and with the wild type; the protein and peptide structure files were uploaded as Molecules 1 and 2, respectively. The active residues that form the interaction of proteins with each other in each complex have been provided according to preliminary information from PDBsum which is one of several online databases that provide information on all experimentally determined structural models published by the Protein Data Bank (PDB) [38]. Rigid molecular docking with flexibility on both active protein side chains and peptide structures was executed using the default settings [38]. The docking score (haddock score) of each variant of TERB1, TERB2, and MAJIN is hereafter designated as the stability index of TERB1-TERB2 and TERB2-MAJIN complexes. Cluster with the lowest haddock score is the most likely conformation [39].

#### 2.7. Binding Affinity Prediction

Subsequently, the protein binding energy prediction (PRODIGY) web server was used to predict the binding affinity of the protein-protein complexes from their 3D structure [39, 40]. Complexes with the lowest energy value (ΔΔ*G*) have greater binding affinity and correlates with mutations that stabilize the protein structures [40].

### 2.8. Analysis of Protein-Protein Interactions

Ligplot software uses the 3D coordinates of a protein and its bound protein/ligand to automatically produce schematic diagrams. These diagrams show the pattern of interactions between the two molecules and are very useful for comparing different structures since they provide clear and helpful information of the intermolecular interactions and their strengths, including hydrogen bonds, hydrophobic interactions, and atom accessibilities [41].

#### 2.9. Assessment of Protein Stability

The stability of the protein was checked using the Protein Stability Change Upon Mutation (DUET), which is an online server for an integrated computational approach to study missense mutations in proteins. It combines two complementary approaches (mCSM and SDM) in a consensus prediction, obtained by consolidating the results of the separate methods in an optimized predictor using Support Vector Machines (SVM) [42]. DUET predicts the change in stability by calculating the change in unfolding free energy (ΔΔ*G*). It further defines whether these changes increase or decrease the stability of the protein. Positive ΔΔ*G* value denotes that protein stability increased, and negative ΔΔ*G* means that protein stability decreased [43].

Site Directed Mutator (SDM) is an algorithm of statistical potential energy function that calculates a stability score based on environment-specific amino acid substitution frequencies within homologous protein families, which is comparable to the free energy difference between wild-type and mutant proteins [44].

Mutation Cutoff Scanning Matrix (mCSM) relies on graph-based signatures used as a novel approach to the study of missense mutations and the prediction of their effects. These signatures encode distance patterns between atoms and are used to represent the protein residue environment and to train predictive models [45].

### 2.10. Phylogenetic Conservational Analysis

To predict the evolutionary conservation of the amino acids in a protein sequence, we used a ConSurf bioinformatic tool that provides evolutionary profiles of each of the amino acids in the protein, based on phylogenetic relations between homologous sequences to reveal regions that are important for structure and/or function [46]. The tool also predicts the conservation score for each amino acid residue ranging from 1 to 9, where the score denotes the degree to which the amino acids are evolutionary conserved: 1–3 designate variable residues, 4–6 designate medium conserved scores, and 7–9 depict highly conserved residue [47, 48].

#### 2.11. Root Mean Square Deviation Calculation

YASARA View is an open-source program for the molecular graphics, modeling, visualization, and analysis of the three-dimensional protein's structures. The structural deviations between the native and mutated models were analyzed using the YASARA View program, by measuring the root mean square deviation (RMSD) which is the average distance between the atoms of the superimposed proteins [49]. Its values are considered to be reliable indicators of variability, where values superior than 0.15 were estimated to be significant and can affect protein function and/or structure [49].

### 2.12. Prediction of Posttranslational Modification Sites

A posttranslational modification is crucial for cell signaling and affects the function of the protein. TERB1, TERB2, and MAJIN proteins' posttranslational modifications were predicted using MusiteDeep, an online service that includes a deep learning framework for predicting protein posttranslational modification (PTM) sites [50].

## 3. Results

### 3.1. SNP Dataset

**Figure 4:**
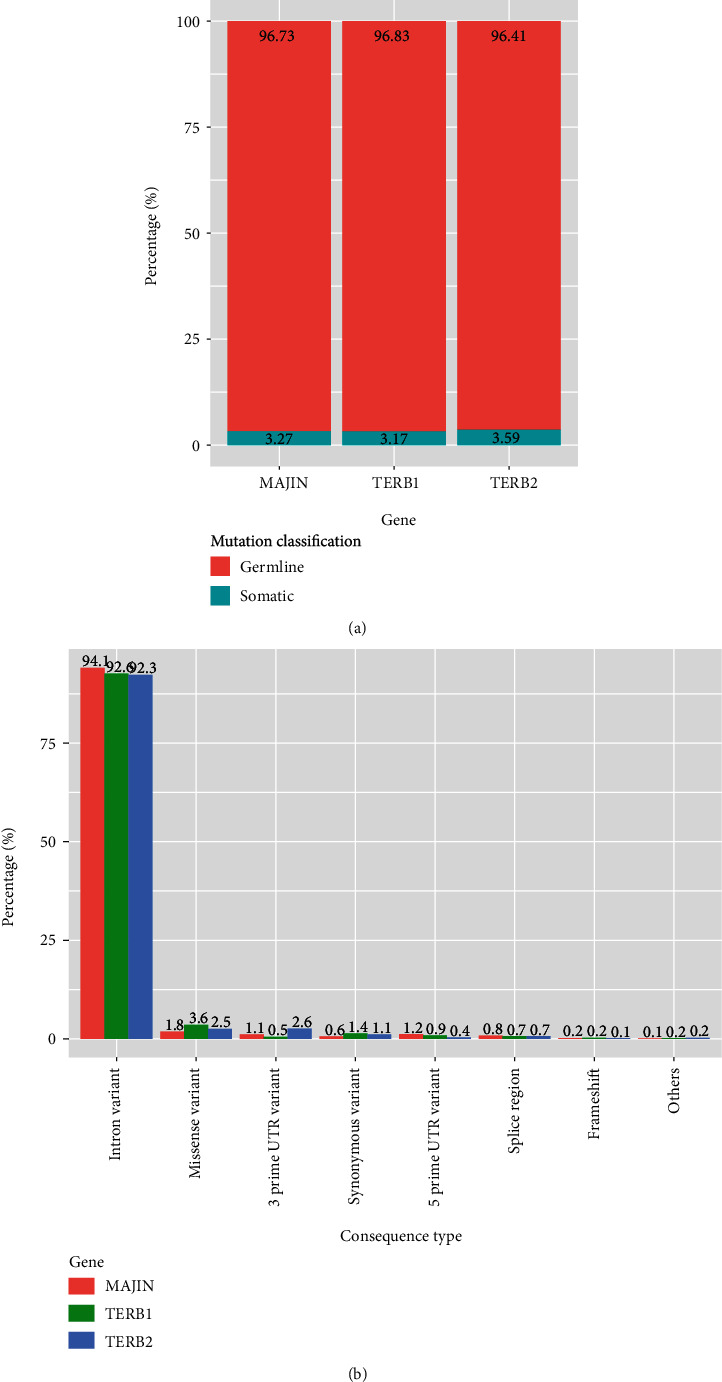
(a) Mutation classification of TERB1, TERB2, and MAJIN proteins. (b) Consequence type of TERB1, TERB2, and MAJIN genes.

The SNP dataset was collected from Ensembl database and had almost the same classification in the three studied proteins: about 96% involved germinal cells, while 3% concerned somatic cells. Different consequence types of these SNPs were detected: intron variant with the higher percentage (TERB1: 92.6%; TERB2: 92.3%; MAJIN: 94.1%), followed by missense variant (TERB1: 3.6%; TERB2: 2.5%; MAJIN: 1.8%), and other variants—3 prime UTR, 5 prime UTR, synonymous, splice region, and frameshift. In this study, seventy missense mutations that could have been mapped on the protein structure were included: 18 variants for TERB1, 31 variants for TERB2, and 21 variants for MAJIN (Figure 4).

### 3.2. Retrieval of Deleterious SNPs

Thirty missense SNPs were predicted to be deleterious using SIFT (where tolerance index score was ranged from 0 to 0.02). The SNPs predicted by SIFT were validated by PolyPhen2, MutationTaster, MetaSVM, and MetaLR. In particular, PolyPhen2 results showed that eight AA substitutions (R620C, I624T, S608C, F607V, R605Q, C592Y, C592R, and L189P) were predicted to be possibly damaging, while twenty-two SNPs were probably damaging: P649A, K632E, Y631S, Y631H, Y631N, H621Q, H621L, C618R, R605G, and S604G in TERB1 gene; W8R, G25R, G25V, F64S, G84S, G84D, P90S, G187W, D191N, and G198R in TERB2 gene; and R53H and R53C in MAJIN gene. The MutationTaster tool classified all these AA substitutions as deleterious (Table 1).

For the purpose of detecting the most deleterious SNPs and to estimate the effects of single mutations on protein-protein interactions, MutaBind2 web server was used. In total, thirty mutations were deleterious with a change in binding free energy ΔΔ*G* ≥ 1.5: seventeen variants for TERB1, eleven variants for TERB2, and two variants for MAJIN (Table 2).

### 3.3. Assessment of Binding Energy and Binding Affinity

**Figure 5:**
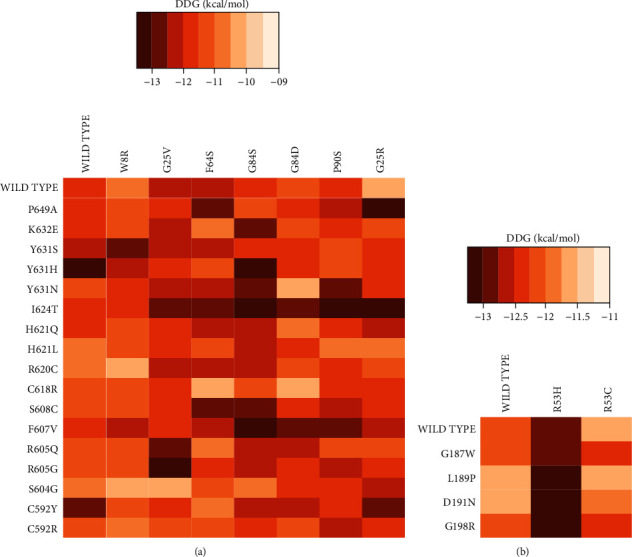
Heatmap of global binding energy score. A: TERB1-TERB2 complex, B: TERB2-MAJIN complex.

For the molecular docking, HADDOCK2.4 web server was used. The top cluster generated by HADDOCK2.4 was considered reliable based on available literature. To ascribe biological relevance to the haddock score, it was further processed with PRODIGY online server to calculate binding energy. Results of PRODIGY showed that the lowest binding energy indicates greater binding affinity, while higher binding energy is giving less binding affinity (Figure 5).

Based on heatmap results, regions with the lightest orange shade represent complexes with higher free binding energy (ΔΔ*G*) which correlates with lower binding affinity. These regions are represented by seven mutant complexes of TERB1-TERB2 complex (R620C-W8R; S604G-W8R; S604G-G25V; C618R-F64S; C618R-G84D; Y631N-G84D; and WT TERB1-G25R) with a ΔΔ*G* ≈ −10 kcal/mol and four complexes of the TERB2-MAJIN complex (WT TERB2-R53C; WT MAJIN-L189P; WT MAJIN-D191N, and L198P-R53C) with a ΔΔ*G* ≈ −11.5 kcal/mol (Figure 5).

### 3.4. Analysis of Protein-Protein Interactions

This step examines protein-protein interactions of complexes to gain a better understanding of the main changes caused by the missense mutations. The comparison between native and mutated proteins in the complexes highlights the differences which may affect their structures, biological functions, or physical properties. The interactions shown by Ligplot program are those mediated by hydrogen bonding and hydrophobic interactions. According to heatmap results, only complexes that showed lower binding affinity were analyzed by Ligplot: R620C-W8R, S604G-W8R, S604G-G25V, C618R-F64S, C618R-G84D, Y631N-G84D, and WT TERB1-G25R of TERB1-TERB2 complex and WT TERB2-R53C, WT MAJIN-L189P, WT MAJIN-D191N, and L198P-R53C of TERB2-MAJIN complex (Table 3).

Ligplot result of the wild-type complex **TERB1-TERB2** showed 19 hydrogen bonds formed between amino acids (Gly, Ala, Val, Glu, Ser (3), Leu (2), Arg (8), Lys, and His) of TERB1 and amino acids (Ser, Leu (2), Lys (2), Thr, Asp (5), Gln, Ala (2), Asn (2), Glu (2), and Ile) of TERB2. In this complex, chain A (TERB2) revealed 12 hydrophobic interactions (Tyr, Arg, Val, Ala, Cys (2), Leu, Phe, Asn, His, Ile, and Glu) while chain B (TERB1) showed 14 hydrophobic interactions (Cys, Leu, Pro, His, Ile (3), Asn, Ala (2), Gly, Phe (2), and Tyr) (Table 3).

Mutated complex represented some differences in the hydrogen bonds and hydrophobic interactions: some contacts were added and others were removed. **R620C-W8R** complex showed that hydrogen bonds formed by Gly, Val, Ser (3), Arg (8), Leu, His, Leu, Ile, Thr, Asp, Asn, and Gln amino acids were deleted, while new bonds with other residues (Asp (5), Asn, Thr (4), Gln, Arg, and His) were added. Further hydrophobic interactions have been added underlying the following Gly, Gln, Val, and Ser amino acids, whereas Ile, Cys, and Asn amino acids were eliminated.

Nine amino acids (Asp, Asn, Thr, Cys, Tyr, Arg, His, Gly, and Tyr) were added to hydrogen bonds of the mutated complex **S604G-W8R**; on the other hand, eight amino acids (Gly, Val, Ser, Arg, Leu, Thr, Asp, and Gln) were removed. Four hydrophobic contacts (Gln, Gly, Val, and Arg) were added, and eight were deleted (Tyr, Arg, Cys, Asn, His, Glu, Pro, and Tyr).

**S604G-G25V** complex showed that hydrogen bonds constituted by Gly, Asp, Asn, Arg, Tyr, Gln, Thr, and His residues were added and Leu, Val, Asp, Glu, Thr, Asn, Arg, Gln, His, Lys, Ser, and Ile were removed. Some hydrophobic interactions involving Gln, Lys, Ser, Arg, Val, and Pro have been added, and Arg, Cys, Asn, Val, Lys, Glu, Pro, and His were eliminated.

The mutated complex **C618R-F64S** revealed that hydrogen bonds formed by Asp, Ile, Asn, Thr, Gly, Gln, Arg, and His amino acids were added, while those formed by Gly, Val, Ser, Arg, Lys, His, Leu, Ile, Ala, Thr, Glu, Asp, and Gln were canceled. For hydrophobic contacts, Lys, Gln, Pro, Val, and Arg amino acids were added and Arg, Cys, Asp, His, Pro, Asn, and Gly amino acids were deleted.

Eight amino acids (Asp, Asn, Tyr, Thr, Gln, Arg, His, and Tyr) were added, and nine (Gly, Ala, Val, Ser, Leu, Arg, Lys, Ile, and Gln) were removed in the hydrogen bonds of the mutant complex **C618R-G84D**. Nine hydrophobic interactions (Arg, Val, Cys, Asn, Glu, Leu, Pro, His, and Tyr) were added, while four (Lys, Pro, Val, and Arg) were removed.

The mutated complex **Y631N-G84D** revealed that hydrogen bonds formed by Asp, Tyr, Asn, Arg, Cys, Ile, His, and Phe amino acids were added and hydrogen bonds formed by Leu, Val, Asp, Glu, Thr, Gly, Ser, Lys, Arg, and Ile amino acids were removed. For hydrophobic contacts, six amino acids (Gln, Pro, Thr, Gly, Val, and Arg) were added while nine amino acids (Tyr, Arg, Val, Cys, Asn, His, Pro, and Phe) were canceled (Table 3).

**WT TERB1-G25R** complex showed that hydrogen bonds formed by Asp, Gln, Tyr, Asn, Thr, Arg, Tyr, and His amino acids were added, while those formed by Val, Ser, Arg, His, Leu, Thr, Asp, Gln, Asn, and Ile amino acids were deleted. Five hydrophobic contacts (Ser, Asp, Glu, Arg, and Val) were added, and five (Arg, Asn, Glu, Pro, and Tyr) were removed.

The second wild complex **TERB2-MAJIN** undergoes 20 hydrogen bonds formed between amino acids (Ser, Lys (5), Arg (6), Phe, His (3), Asn, Ala (2), and Gly) of MAJIN chain and amino acids (Leu (3), Asp (3), Phe (3), His (2), Tyr (3), Gly (3), Lys, and Ile (2)) of TERB2 chain. MAJIN protein carries 19 hydrophobic contacts (Leu (5), Ser (3), Phe (3), Ile, Ala, Thr, Val, Asn, Gln, Asp, and Gly) while 13 hydrophobic interactions (Gly (2), Ala, Val, Ile, Glu, Tyr, Met, Lys (2), Leu (2), and Ser) were detected in TERB2 protein.

The mutated complex **WT TERB2-R53H** showed that six amino acids (Ser, Asp, Cys, Gly, Val, and Lys) were added to hydrogen bond interactions, while three (Arg, Gln, and Pro) were removed. The hydrophobic interactions listed two amino acids (Ile and Asp) as deleted and only one amino acid (Pro) as added.

Five amino acids (Arg, Ala, Gly, Ile, and Val) were added to hydrogen bonding of the mutated complex **MAJIN-L189P**, and five were removed (Pro, Gln, Ala, Ser, and Ile). Hydrophobic contacts added three new residues (His, Gln, and Pro) to the list, while four residues Leu, Ile, Val, and Gly were taken off (Table 3). The mutated complex **MAJIN-D191N** showed that hydrogen bonds formed by Ala, Val, Asp, and Lys amino acids were dismissed, while two others were added (Leu and Asp). Some hydrophobic interactions implying Gly, His, and Asn have been added, and Asp, Val, and Ile have been eliminated (Table 3). About the hydrophobic contacts of the mutant complex **L189P-R53C**, two amino acids were added (Gln and Pro), and six were canceled (Ile, Asp, Gly, Glu, Leu, and Ser). In the hydrogen bonds, six amino acids (Asp, Gly, Cys, Glu, Gly, and Lys) were added, while one (Gln) was removed.

DUET, ConSurf, and MusiteDeep web servers were used to identify, respectively, the mutation effects on protein stability and the conserved domains in proteins and predict directly the posttranslational modification (PTM) site from the raw protein sequence. The mutated structures were also analyzed by YASARA View, with regard to evaluate conformational variations by calculating the RMSD.

### 3.5. Effect on the Protein Stability

The results of mCSM, SDM, and DUET algorithms showed that 23 amino acids substitutions were recorded as destabilizing by the three algorithms: P649A, K632E, Y631S, Y631H, Y631N, I624T, H621Q, C618R, F607V, R605Q, R605G, C592Y, and C592R of TERB1; W8R, F64S, G84S, G84D, P90S, G25R, G187W, L189P, and G198R of TERB2; and R53C of MAJIN (Table 4). Furthermore, other mutations increased the stability of the proteins by one or two algorithms (Table 4).

#### 3.6. Phylogenetic Conservation Analysis of High-Risk SNP

Compared to those in nonconserved regions, amino acids located in conserved regions were predicted to be highly damaging. ConSurf predicts amino acids to play structural or functional roles based on conservation and solvent accessibility. Residues are predicted as functional when they are highly conserved and exposed and as structural when they are highly conserved and buried.

**Figure 6:**
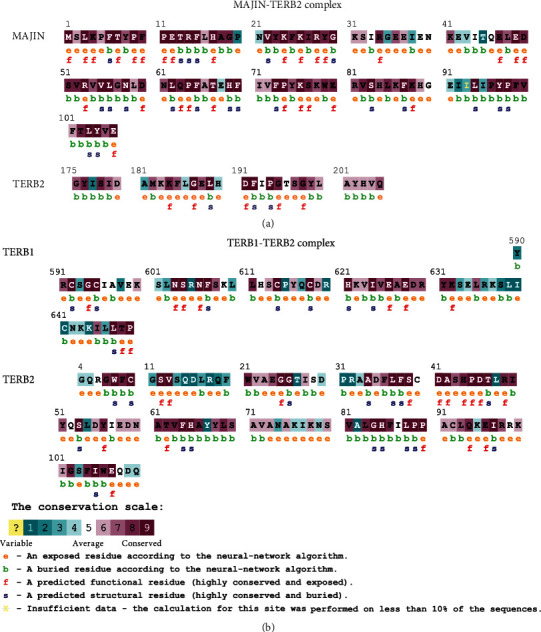
Results of ConSurf prediction of TERB1, TERB2, and MAJIN proteins. (a) TERB2-MAJIN complex. (b) TERB1-TERB2 complex.

Phylogenetic conservation analysis of TERB2 protein showed that G25, P90, G187, D191, and G198 are highly conserved residues and predicted to be exposed and functional. On the other hand, W8, F64, G84, and L189 are highly conserved residues and predicted to be buried and structural. Finally, R53 is highly conserved and predicted to be exposed and a functional residue in MAJIN protein (Figure 6(a)).

Analysis of TERB1 protein from the ConSurf server revealed that P649 and S604 are highly conserved residues and predicted to be exposed and functional, while I624, H621, C618, F607, and C592 are highly conserved residues and predicted to be buried and structural. K632, R620, S608, and R605 are exposed residues: R620 is variable, while K632 and S608 are conserved. Y631 is a conserved and buried residue (Figure 6(b)).

#### 3.7. Root Mean Square Deviation (RMSD) Prediction

**Figure 7:**
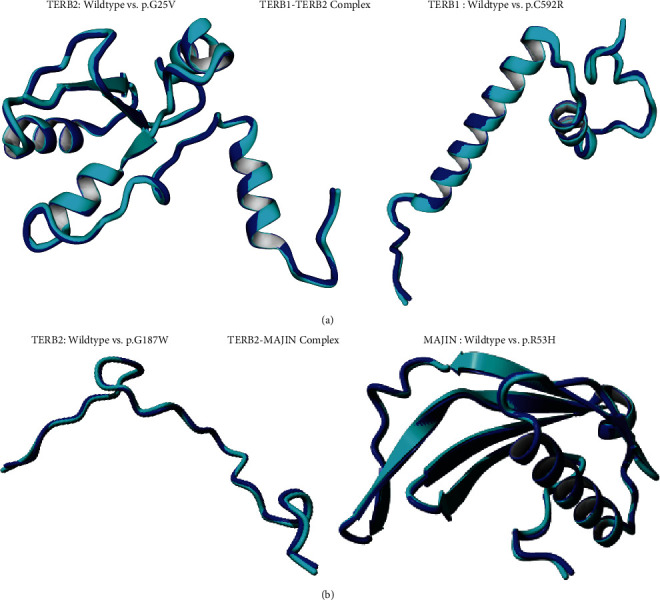
Superimposed 3D structures of the wild (Cyan) and highly deleterious mutated proteins (Blue). (a) TERB1-TERB2 complex. (b) TERB2-MAJIN complex.

In our study, 29 mutations out of 30 showed higher RMSD values which indicate greater variation between wild and mutant protein structures. Therefore, four variants C592R, G25V, G198R, and R53H reflected maximal structural dissimilarity with TERB1-TERB2 complex and TERB2-MAJIN complex (1.823, 1.4844, 1.4723, and 1.6674, respectively) and were therefore used for superimposing (Figure 7). One residue change G25R in TERB2 protein showed a RMSD value equal to 0 suggesting a minimal structural deviation between the native model and this mutation (Table 5).

### 3.8. Prediction of Posttranslational Modification Sites

MusiteDeep is applied to predict the effect of SNPs on posttranslational modification (PTM) process of the human TERB1, TERB2, and MAJIN proteins. MusiteDeep identified sites for O-linked glycosylation: R605 in TERB1 protein and W8, G84, G187, L189, D191, and G198 in TERB2 protein sequence (Table 6). S-Palmitoylation site was identified in C592 of TERB1 protein. No PTM site has been located on MAJIN protein sequence.

### 3.9. Cumulative Score (CS) Calculation

A cumulative score (CS) was calculated for the 30 damaging variants based on seven in silico tools used for predicting the effect of SNPs (SIFT, PolyPhen2, Mutabind2, Prodigy, stability by SDM, mCSM, DUET, and RMSD estimated by YASARA, and ConSurf) to further understand and justify how the most deleterious mutations were identified in this study (Table 7).

All of the listed 30 SNPs were predicted as deleterious or damaging by SIFT (SIFT < 0.05), PolyPhen2 (possibly and probably damaging SNPs), and Mutabind2 (ΔGG ≥ 1.5) (Table 7); and only eleven were considered highly pathogenic SNPs since they were agreed significantly by tools with the following scores: Prodigy's ΔGG ≥ –11 kcal/mol; SDM, mCSM, and DUET's change in unfolding free energy ΔGG ≤ 0; RMSD values ≥ the median (1.65 for TERB1-TERB2 complex) and (1.47 for TERB2-MAJIN Complex); and a conservation score = 9.

## 4. Discussion

The results of several recently published papers have contributed to our understanding of the meiotic telomere complex assembly in mammals and its possible interaction with Shelterin and LINC complexes, thus providing a significant advance in the identification of the importance of meiotic telomeres complex which has been gradually elucidated and clarified by various authors. In 2014, Shibuya et al. showed that TERB1 = CCDC79 structure contains two armadillo repeats at the N terminus, a coiled coil motif in the middle region and, remarkably, a Myb domain at the C terminus, a structure which resembles that of TRF1. At this point, many roles were dedicated to TERB1 [21]. It is important for homologue pairing/synapsis and recombination and for male and female fertility. TERB1 assembles a large protein complex linking telomeres to the SUN1-KASH5 complex along the nuclear envelope in meiosis. Moreover, TERB1 binds to telomeres by forming a heterocomplex with TRF1, and its Myb domain performs a crucial role in enriching cohesin, thereby promoting the structural integrity of telomeres during prophase I [21].

In addition to TERB1, two downstream meiotic proteins, TERB2 and MAJIN, are recruited to the meiotic telomeres by TERB1 in mammalian meiosis [22]. Shibuya et al. affirmed that MAJIN was the key player, named also after a Japanese word representing the “Genie in Aladdin's Lamp.” MAJIN behaves as a membrane protein and sequesters telomere adaptors TERB1/2 to the INM. Thus, they concluded that TERB2-MAJIN plays a critical role in assembling SUN1-KASH5 at telomeres and achieving meiotic chromosome movement [22]. In 2017, Long et al. focused on the various key domains of TERB1 that mediate different protein interactions that are required for constructing meiosis-specific telomere structures that enable telomere attachment and movement along the NE for faithful homolog pairing in mammalian meiosis [4]. One year later, Dunce et al. reported the crystal structure of the MAJIN-TERB2 complex, revealing a 2 : 2 heterotetramer in which two TERB2 chains wrap around a core MAJIN globular dimer. This structure endures direct interaction with DNA and scaffolds assembly of the full 2 : 2 : 2 TERB1-TERB2-MAJIN of the meiotic telomere complex, which can recruit two TRF1 dimers. Together, this data confirmed that TRF1-mediated loading of telomeric DNA to the meiotic DNA leads to a molecular model in which a hierarchical succession of binding processes achieves meiotic telomere attachment [3].

In 2019, Wang et al. concluded that the intact MTC complex is required for gametogenesis and fertility, and targeted disruption of the complex induces disordered synapsis and impairs meiotic double-strand break (DSB) repair and abolishes telomere-NE linkage, together leading to a complete meiotic arrest in prophase I in both sexes. Hence, both the MTC complex and SUN1 (the LINC complex) contribute to the stable telomere-NE association and are important for efficient progression of prophase I [24].

Last year, Salas-Huetos et al. strongly confirmed that disruption of any of the three genes coding for the meiotic telomere complex (MTC) can cause NOA in men. It has been demonstrated previously that disruption of Terb1 in the mouse results in meiotic arrest and impairment of homologous pairing and synapsis, ultimately resulting in infertility in both males and females [21]. Likewise, mice disrupted for either MAJIN or TERB2 display impaired synapsis, zygotene arrest, a lack of post-meiotic cells, and infertility phenotype as revealed by Salas-Huetos [51].

Taken together, they showed that the disruption or mutations of any of the MTC genes impairs telomere association with the inner nuclear membrane, which is requisite for chromosomal synapsis and chromosome movement during meiosis. Failure of these events triggers meiotic checkpoints leading to a meiotic arrest and consequently to male infertility/NOA [51]. The main goal of this work was exploring the effects of missense mutations on meiotic telomere complex proteins (TERB1, TERB2, and MAJIN) related to male infertility.

Bioinformatic analysis provides powerful insights into understanding and predicting the effects of mutations on protein structures, including how mutations alter protein stability, binding energy, binding affinity, evolutionary conservation and interactions with other proteins and their relation to susceptibility of genetic diseases. In the current study, various algorithms, namely, VEP tools (SIFT, PolyPhen2, and MutationTaster), Mutabind2, Haddock, Prodigy, Ligplot, DUET, ConSurf, and MusiteDeep, were used to identify the most deleterious SNPs of the TERB1, TERB2, and MAJIN genes. Accordingly, eleven SNPs out of thirty (W8R, G25R, P649A, I624T, C618R, F607V, S604G, C592Y, C592R, G187W, and R53C) scored the highest cumulative scores (CS = 6) predicted by different bioinformatic tools and were located in conserved regions with a score conservation of 9, which may affect the structure and function of proteins (Table 7).

Protein-protein docking aims to anticipate molecular complex's structure given the individual solved structures of its constituents, which can be determined experimentally or predicted. In the present study, docking was performed by HADDOCK web server that displays results as cluster's structures, where the first top cluster produced is deemed reliable and should be downloaded in order to be loaded on the PRODIGY web server to calculate binding energy. Binding affinity refers to the strength or interaction energy of the binding between the protein and its partner. According to the results of heatmap, the lighter colored areas represent the complexes with higher binding energy and less binding affinity: R620C-W8R, S604G-W8R, S604G-G25V, C618R-F64S, C618R-G84D, Y631N-G84D, and WT TERB1-G25R of TERB1-TERB2 complex and WT TERB2-R53C, WT MAJIN-L189P, WT MAJIN-D191N, and L198P-R53C) of TERB2-MAJIN complex (Figure 5). Generally, the steady state of a protein is the state with the lowest energy level. The energy score influences protein's stability and binding affinity which means: lower energy value correlates with a favorable mutation that stabilizes the protein structure and can potentially improve binding affinity, and higher energy values refer to mutations that decrease protein stability and potentially alter the binding affinity.

Missense mutations can affect protein-protein interactions via various molecular mechanisms, including changing folding free energy of interacting partners, modifying protein stability, or disrupting noncovalent interactions essential for complex formation and function. Binding affinity is also influenced by noncovalent interactions between biomolecules, such as the loss or gain of hydrogen bonding and/or hydrophobic contacts in the mutated complex compared to the wild-type complex. After visualization by Ligplot, we concluded that the eleven previous complexes with the lower binding affinity showed differences in protein-protein interactions where some contacts were added and others were deleted, which explains the diminution of binding affinity within these complexes (Table 3).

DUET online server was used to check the significant effect of mutations on the stability of protein structure. Out of the most deleterious variants, ten variants revealed negative unfolding free energy (ΔΔ*G*) predicted by the 3 algorithms which indicate that they destabilized the protein's stability, except for the S604G that was destabilizing only by two algorithms (Table 4).

The RMSD computation was used to assess the superposition when comparing native and mutated proteins. Among the eleven mutations, ten showed higher RMSD values, except for the residue change G25R that had a RMSD value = 0 indicating (Table 5).

Conservation analysis is important in unraveling whether the SNPs are found in a conserved region or not. Binding regions are known to be evolutionarily conserved, which has been evaluated in various studies to identify potential protein interaction interfaces. Thus, according to Miller and Kumar, highly conserved amino acids are located in biologically active sites. When these residues are substituted, biological activities are affected [52]. The results of the ConSurf analysis showed that the eleven amino acid changes (W8R, G25R, P649A, I624T, C618R, F607V, S604G, C592Y, C592R, G187W, and R53C) were located in conserved regions (Figure 6).

MusiteDeep identified sites for O-linked glycosylation W8 and G187 in TERB2 protein sequence with a score of 0.864 and 0.771, respectively. Consequently, mutations at these positions might affect function and/or structure of the TERB2 protein (Table 6).

Most improvements to computational predictions of mutation effects have been achieved by the identification of SNPs and their impact on human health, which has been the most explored field in human genetics recently. In this study, we applied several tools to prioritize damaging SNPs and estimated the most deleterious ones based on binding energy and affinity evaluation, stability assessment, evolutionary conservation analysis, and posttranslational modification site prediction. However, in silico prediction of protein-protein interaction network in native and wild protein should be confirmed with extensive experiments and lab approaches to figure out the mechanism and impact of these mutations in susceptibility to male infertility.

## 5. Conclusion

In the current study, out of 32 missense mutations, 11 were cumulatively predicted to be high risk pathogenic SNPs (W8R, G25R, P649A, I624T, C618R, F607V, S604G, C592Y, C592R, G187W, and R53C) as they were agreed by six software. Combinations of multiple in silico tools provided information required to predict the effects of these mutations on the functional and structural deviations of TERB1, TERB2, and MAJIN proteins. This work has highlighted the need to take into account the type of protein-protein interactions when characterizing the variants within the complexes. Hence, our result added to previous knowledge and supported earlier findings that may be helpful for further understanding the role of meiotic telomere complex (MTC) in male infertility. Nevertheless, bioinformatics tools cannot replace conclusive experiments, and their results should be verified by supplementary data.

## Figures and Tables

**Table 1:** The list of selected pathogenic SNPs out of 70 variants using VEP tools.

Protein	SNP	Substitution	SIFT		PolyPhen2		MutationTaster		MetaSVM		MetaLR	
			P	S	P	S	P	S	P	S	P	S
TERB1	rs182898829	P649A	del	0	Prob Dam	0.996	D	0.984103	T	-0.3046	T	0.375
	rs1271099093	K632E	del	0	Prob Dam	0.923	D	0.915046	T	-0.2958	T	0.3641
	rs1485173952	Y631S	del	0	Prob Dam	0.997	D	0.943291	T	-0.2165	T	0.3981
	rs1185337560	Y631H	del	0.02	Prob Dam	0.998	D	0.768475	T	-0.2165	T	0.3981
	rs1185337560	Y631N	del	0	Prob Dam	0.998	D	0.973837	T	-0.2165	T	0.3981
	rs1451941518	I624T	del	0	Pos Dam	0.674	D	0.704276	T	-0.5983	T	0.2435
	rs1376123957	H621Q	del	0	Prob Dam	0.997	D	0.865243	T	-0.4454	T	0.3198
	rs1476862679	H621L	del	0	Prob Dam	0.997	D	0.998697	T	-0.2165	T	0.3981
	rs192051533	R620C	del	0.02	Pos Dam	0.72	D	0.666354	T	-0.7483	T	0.1868
	rs577058590	C618R	del	0	Prob Dam	0.998	D	1	T	-0.2165	T	0.3981
	rs363181	S608C	del	0	Pos Dam	0.819	D	0.567768	T	-0.465	T	0.2944
	rs779131904	F607V	del	0	Pos Dam	0.626	D	0.988206	T	-0.5444	T	0.2559
	rs1280669541	R605Q	del	0	Pos Dam	0.571	D	0.852851	T	-0.5746	T	0.2315
	rs963945421	R605G	del	0	Prob Dam	0.97	D	0.93175	T	-0.4836	T	0.3139
	rs1346512957	S604G	del	0	Prob Dam	0.912	D	0.984073	T	-0.2738	T	0.3709
	rs1056107219	C592Y	del	0	Pos Dam	0.548	D	0.987462	T	-0.5569	T	0.2409
	rs760907067	C592R	del	0	Pos Dam	0.548	D	0.999999	T	-0.6064	T	0.2289
TERB2	rs1287250231	W8R	del	0	Prob Dam	0.974	D	0.752306	T	-0.1875	T	0.3554
	rs780054329	G25R	del	0	Prob Dam	1	D	0.985902	T	-0.635	T	0.2366
	rs371121909	G25V	del	0	Prob Dam	1	D	0.999999	T	-0.6471	T	0.2366
	rs752515965	F64S	del	0	Prob Dam	0.994	D	0.890869	T	-0.3724	T	0.2896
	rs1362128332	G84S	del	0	Prob Dam	0.998	D	0.953812	T	-0.5932	T	0.2309
	rs374008371	G84D	del	0	Prob Dam	0.999	D	0.98029	T	-0.752	T	0.1798
	rs376927152	P90S	del	0	Prob Dam	0.996	D	0.922359	T	-0.6591	T	0.1975
	rs183491207	G187W	del	0.02	Prob Dam	1	D	0.964209	T	-0.5958	T	0.227
	rs1566947814	L189P	del	0	Pos Dam	0.82	D	0.999997	T	-0.7824	T	0.1813
	rs1266359742	D191N	del	0.02	Prob Dam	0.99	D	0.70784	T	-0.5641	T	0.1924
	rs1195151997	G198R	del	0	Prob Dam	0.999	D	0.942803	T	-0.6662	T	0.1957
MAJIN	rs375342082	R53H	del	0	Prob Dam	0.999	D	0.936673	T	-0.1519	T	0.3977
	rs377370396	R53C	del	0	Prob Dam	1	D	0.999326	T	-0.419	T	0.3252

AA: amino acid; P: prediction; S: score; Del: deleterious; Prob Dam: probably damaging; Pos Dam: possibly damaging; T: Tolerated.

**Table 2:** Summary of the most deleterious Mutations generated by Mutabind2 for TERB1-TERB2 complex and TERB2-MAJIN complex.

Complex	Protein	Mutation	ΔΔ*G*_bind_	Deleterious	ΔΔ*E*_vdw_	ΔΔ*G*_solv_	ΔΔ*G*_fold_
TERB1-TERB2 complex	TERB2	W8R	1.59	Yes	-0.0986	0.6838	0.0997
		G25R	1.62	Yes	-0.121	0.5061	0.8582
		G25V	1.67	Yes	-0.1415	0.4611	0.8399
		F64S	2.24	Yes	-0.2398	0.4953	0.8775
		G84S	2.45	Yes	0.4805	0.3867	0.8418
		G84D	3.39	Yes	0.492	2.0154	0.7326
		P90S	2.04	Yes	-0.0947	0.5128	0.7821
	TERB1	S608C	1.6	Yes	-0.2338	0.6287	-0.3566
		F607V	2.69	Yes	0.0063	0.4449	0.8964
		R605Q	2.19	Yes	0.0004	0.852	-0.2899
		R605G	2.77	Yes	0.2268	1.1856	-0.3705
		S604G	3.02	Yes	-0.352	0.4639	0.8462
		C592Y	1.91	Yes	-0.1474	0.6433	-0.2039
		C592R	1.74	Yes	-0.118	0.627	-0.3644
		P649A	1.7	Yes	-0.1537	0.7697	-0.2818
		K632E	3.18	Yes	0.6997	0.6296	1.0157
		Y631S	2.78	Yes	-0.2333	0.5476	0.8309
		Y631H	2.24	Yes	-0.3086	0.6727	0.4667
		Y631N	2.58	Yes	-0.4575	0.5622	0.8561
		I624T	2.68	Yes	-0.1331	0.8786	0.8066
		H621Q	2.33	Yes	-0.0866	0.6944	-0.2951
		H621L	2.15	Yes	-0.2391	0.6956	-0.2847
		R620C	1.8	Yes	-0.1548	0.7717	-0.2466
		C618R	1.58	Yes	-0.1822	0.7123	-0.389
TERB2-MAJIN complex	MAJIN	R53H	2.16	Yes	-0.1919	0.4687	0.9622
		R53C	1.72	Yes	-0.3041	0.4805	0.8639
	TERB2	G187W	2.04	Yes	-0.281	-0.0214	0.9273
		L189P	4.27	Yes	-0.259	0.3102	1.5636
		D191N	2.31	Yes	-0.3749	0.476	0.1069
		G198R	1.86	Yes	-0.3637	0.1059	1.0742

**Table 3:** Summary of protein-protein interactions of TERB1-TERB2 complex and MAJIN-TERB2 complex by Ligplot.

Protein interaction	Hydrogen bonds		Hydrophobic interactions	
TERB1-TERB2 complex	Chain A	Chain B	Chain A	Chain B
TERB1-TERB2 (wild type)	Gly, Ala, Val, Glu, Ser (3), Leu (2), Arg (8), Lys, His	Ser, Leu (2), Lys (2), Thr, Asp (5), Gln, Ala (2), Asn (2), Glu (2), Ile	Tyr, Arg, Val, Ala, Cys (2), Leu, Phe, Asn, His, Ile, Glu	Cys, Leu, Pro, His, Ile (3), Asn, Ala (2), Gly, Phe (2), Tyr
R620C-W8R	Ala, Asp (5), Leu (2), Glu (3), Asn, Thr (4), Lys, Gln	Arg (6), Ser (6), Lys (2), His, Ala, Glu (2)	Leu (2), Cys (2), Val (2), Ala, Glu, Tyr, Asn, Arg, Phe, Gly, His	Gln, Ile (4), Tyr (2), Leu (2), Ala (3), Pro, Phe (2), His, Gly, Val, Ser
S604G-W8R	Ala, Asp (4), Lys (2), Glu (2), Asn (2), Thr (2), Leu, Cys, Tyr, His	Arg (4), Ser, Lys (3), His (2), Ala, Glu (2), Gly, Tyr	Leu, Val, Ala (3), Gln (2), Phe (2), Ser, Gly, Ile (3)	Leu (2), Cys, Val (2), Ala, Gly, Asn, Arg (2), Phe, Ile
S604G-G25V	Ala, Asp (4), Tyr, Asn, Thr (3), Leu, Gly, Gln (2)	Arg (2), Gly (3), Glu (2), Lys (3), His (2), Ser, Ala	Gln, Tyr, Ile (4), Lys, Leu, Pro, Ala (2), Phe (2), Ser, His	Leu (2), Arg (2), Val (2), Ala, Ile, Tyr, Asn, Phe, Gly, Cys
C618R-F64S	Ala (3), Asp (7), Leu, Ile, Asn, Thr (2), Gly (2), Gln (2)	Arg (6), Ser (5), Asn, Lys (3), His (2), Gly (2)	Ala (2), Leu (2), Ile (3), Lys, Tyr (2), Gln, Val, Pro, Phe, Glu	Ala, Cys, Phe, Leu (3), Val (2), Arg, Ile, Tyr
C618R-G84D	Asp (5), Asn (2), Tyr, Thr, Glu, Gln (3)	Arg (4), Ser (2), Lys (4), His, Glu (2), Tyr	Ala (2), Leu (2), Ile (3), Lys, Tyr, Phe (2), His, Pro	Ala, Gly, Cys, Phe, Val, Arg, Ile, Asn
Y631N-G84D	Ala (3), Asp (8), Tyr (2), Asn, His, Cys, Ile	Arg (4), Ser (3), Lys (3), Glu, Phe His, Ala, Cys, Asn	Gln, Leu, Ile (3) Pro, Glu, Ala, Thr	Leu, Ala, Arg (2), Val (2), Gly, Asn, Ile (2)
WT TERB1-G25R	Ala (3), Asp (4), Gln, Tyr, Glu (3), Asn (2), Thr, Leu, Gly, Lys	Arg (8), Ser (3), Tyr, Glu (2), Lys (2), Ala, His	Ile (4), Val, Ser, Ala (2), Leu, His, Tyr, Cys, Phe (2)	Asp, Glu, Arg, Val (2), Ile, Cys, Gly, Ala, Phe, Leu, His
MAJIN-TERB2 complex	Chain C	Chain D	Chain C	Chain D
MAJIN-TERB2 (wild type)	Lys (5), Arg (3), Phe, His (7), Ala (2), Pro	Ser, Leu (2), Phe (2), His (4), Asp (3), Gln, Ile (3), Tyr (3)	Leu (5), Ser (3), Phe (3), Ile, Ala, Thr, Val, Asn, Gln, Asp, Gly	Gly (2), Ala, Val, Ile, Glu, Tyr, Met, Lys (2), Leu (2), Ser
WT TERB2-R53C	Ser, His (5), Ala (2), Lys (5), Asp, Cys, Phe	Leu (2), His (3), Ser, Asp, Tyr (2), Gly, Val, Ile, Phe (2), Lys	Phe (2), Val (3), Leu (5), Ala, Ser, Pro, Gln, Thr, Asn (2), Gly	Ala, Tyr (2), Ile, Leu, Ser, Met, Lys
WT MAJIN-L189P	His (5), Ile (2), Lys (6), Arg, Phe (2), Gly	Leu (2), His (4), Asp (3), Tyr (2), Ala (2), Phe, Arg, Val, Gly	Leu (5), His, Phe (3), Ser, Ile, Ala, Thr, Val, Asn (2), Gln, Asp, Pro, Gly	Gln, Ala, Tyr (2), Ser (2), Met, Lys, Pro, Glu
WT MAJIN-D191N	Leu, His (7), Ala (2), Asp, Lys (3), Phe, Arg, Pro	Gln, Val, Tyr (2), Ile (3), Leu, His (4), Ser, Lys, Phe (3)	Leu (5), Phe (3), Ser, Ala, Thr, Val, Asn, Gln, Asn, Gly (2)	Leu (2), His, Ser, Ala, Tyr (2), Asn, Glu, Gly, Met, Lys
L189P-R53C	His (5), Phe, Ala, Asp, Lys (5), Arg, Gly (2), Pro, Cys	Leu (2), His (4), Ser, Tyr (2), Glu, Gly, Lys, Phe (2), Asp (2), Ile (2)	Phe (3), Leu (5), Ala, Asn, Ser (2), Gln, Thr, Val	Gln, Val, Tyr (2), Ile (2), Lys, Pro, Met, Ala

**Table 4:** Prediction of SNP effects on protein stability using DUET web server.

Complex	Protein	Mutation	mCSM		SDM		DUET	
			ΔΔ*G*	Prediction	ΔΔ*G*	Prediction	ΔΔ*G*	Prediction
TERB1-TERB2	TERB2	W8R	-0.98	Destabilizing	-0.58	Destabilizing	-0.886	Destabilizing
		G25V	-0.041	Destabilizing	-0.52	Destabilizing	0.19	Stabilizing
		F64S	-1.96	Destabilizing	-3.58	Destabilizing	-2.293	Destabilizing
		G84S	-1.273	Destabilizing	-2.09	Destabilizing	-1.298	Destabilizing
		G84D	-1.639	Destabilizing	-2.35	Destabilizing	-1.763	Destabilizing
		P90S	-3.166	Destabilizing	-1.76	Destabilizing	-3.344	Destabilizing
		G25R	-0.589	Destabilizing	-1.92	Destabilizing	-0.584	Destabilizing
	TERB1	P649A	-0.747	Destabilizing	0.0	Destabilizing	-0.448	Destabilizing
		K632E	-1.845	Destabilizing	-0.55	Destabilizing	-1.718	Destabilizing
		Y631S	-3.495	Destabilizing	-2.92	Destabilizing	-3.658	Destabilizing
		Y631H	-2.012	Destabilizing	-0.93	Destabilizing	-1.941	Destabilizing
		Y631N	-3.234	Destabilizing	-2.04	Destabilizing	-3.313	Destabilizing
		I624T	-2.641	Destabilizing	-2.18	Destabilizing	-2.786	Destabilizing
		H621Q	-0.964	Destabilizing	-1.14	Destabilizing	-0.97	Destabilizing
		H621L	-0.205	Destabilizing	1.62	Stabilizing	0.236	Stabilizing
		R620C	0.273	Stabilizing	-0.5	Destabilizing	0.236	Stabilizing
		C618R	-1.641	Destabilizing	-1.16	Destabilizing	-1.514	Destabilizing
		S608C	-0.894	Destabilizing	1.23	Stabilizing	-0.323	Destabilizing
		F607V	-1.319	Destabilizing	-2.51	Destabilizing	-1.578	Destabilizing
		R605Q	-1.027	Destabilizing	-0.9	Destabilizing	-1.116	Destabilizing
		R605G	-1.133	Destabilizing	-0.68	Destabilizing	-1.219	Destabilizing
		S604G	-1.816	Destabilizing	0.31	Stabilizing	-1.704	Destabilizing
		C592Y	-1.69	Destabilizing	-1.68	Destabilizing	-2.034	Destabilizing
		C592R	-1.821	Destabilizing	-1.51	Destabilizing	-1.8	Destabilizing
TERB2-MAJIN	MAJIN	R53H	-2.309	Destabilizing	0.09	Stabilizing	-2.097	Destabilizing
		R53C	-2.108	Destabilizing	-0.69	Destabilizing	-2.066	Destabilizing
	TERB2	G187W	-0.977	Destabilizing	-0.1	Destabilizing	-0.813	Destabilizing
		L189P	-1.568	Destabilizing	-4.23	Destabilizing	-2.238	Destabilizing
		D191N	-0.893	Destabilizing	-0.02	Stabilizing	-0.554	Destabilizing
		G198R	-0.502	Destabilizing	-2.68	Destabilizing	-0.61	Destabilizing

**Table 5:** RMSD calculation results for TERB1, TERB2, and MAJIN proteins.

Complex	Protein	Residue change	RMSD (Å)
TERB1-TERB2 complex	TERB1	P649A	1.6673
		K632E	1.6505
		Y631S	1.6653
		Y631H	1.6745
		Y631N	1.6021
		I624T	1.6776
		H621Q	1.6454
		H621L	1.6412
		R620C	1.6746
		C592R	1.823
		C592Y	1.8092
		F607V	1.7138
		C618R	1.7284
		S604G	1.6745
		S608C	1.6571
		R605Q	1.5727
		R605G	1.5919
	TERB2	W8R	1.3457
		G25V	1.4844
		G25R	0
		F64S	1.3874
		G84S	1.4024
		G84D	1.4118
		P90S	1.4729
TERB2-MAJIN complex	TERB2	G187W	1.4723
		L189P	1.4521
		D191N	1.4415
		G198R	1.4687
	MAJIN	R53H	1.6674
		R53C	1.5528

**Table 6:** MusiteDeep analysis for posttranslational modification sites (PTMs).

Protein	Modification	Residue	Score
TERB1	O-linked glycosylation	R605	0.589
	S-Palmitoylation	C592	0.675
TERB2	O-linked glycosylation	W8	0.864
		G84	0.777
		G187	0.771
		L189	0.782
		D191	0.753
		G198	0.759

**Table 7:** Cumulative score prediction of possible high risk pathogenic SNPs.

Complex	Mutations	SIFT	PolyPhen2	Mutabind2	Prodigy (*Δ*GG)	Stability	RMSD	ConSurf score	Cumulative score (CS)
TERB1-TERB2	W8R	1	1	1	1	1	0	1	6
	G25V	1	1	1	0	0	0	1	4
	G25R	1	1	1	1	1	0	1	6
	F64S	1	1	1	0	1	0	1	5
	G84S	1	1	1	0	1	0	1	5
	G84D	1	1	1	0	1	0	1	5
	P90S	1	1	1	0	1	0	1	5
	P649A	1	1	1	0	1	1	1	6
	K632E	1	1	1	0	1	1	0	5
	Y631S	1	1	1	0	1	1	0	5
	Y631H	1	1	1	0	1	1	0	5
	Y631N	1	1	1	0	1	0	0	4
	I624T	1	1	1	0	1	1	1	6
	H621Q	1	1	1	0	1	0	1	5
	H621L	1	1	1	1	0	0	1	5
	R620C	1	1	1	1	0	1	0	5
	C618R	1	1	1	0	1	1	1	6
	S608C	1	1	1	0	0	1	0	4
	F607V	1	1	1	0	1	1	1	6
	R605Q	1	1	1	1	1	0	0	5
	R605G	1	1	1	0	1	0	0	4
	S604G	1	1	1	1	0	1	1	6
	C592Y	1	1	1	0	1	1	1	6
	C592R	1	1	1	0	1	1	1	6
TERB2-MAJIN	R53H	1	1	1	0	0	1	1	5
	R53C	1	1	1	0	1	1	1	6
	G187W	1	1	1	0	1	1	1	6
	L189P	1	1	1	0	1	0	1	5
	D191N	1	1	1	0	1	0	1	5
	G198R	1	1	1	0	1	0	0	4

## Data Availability

All data are available from the corresponding author upon request.
